# Evolution of mitotic spindle behavior during the first asymmetric embryonic division of nematodes

**DOI:** 10.1371/journal.pbio.2005099

**Published:** 2018-01-22

**Authors:** Aurore-Cécile Valfort, Caroline Launay, Marie Sémon, Marie Delattre

**Affiliations:** 1 Department of Pharmacology & Physiology (Colin Flaveny lab), Saint Louis University School of Medicine, Saint Louis, Missouri, United States of America; 2 UnivLyon, ENS de Lyon, Univ Claude Bernard, Laboratory of Biology and Modelling of the Cell, Lyon University, Lyon, France; Institut Curie, France

## Abstract

Asymmetric cell division is essential to generate cellular diversity. In many animal cells, the cleavage plane lies perpendicular to the mitotic spindle, and it is the spindle positioning that dictates the size of the daughter cells. Although some properties of spindle positioning are conserved between distantly related model species and different cell types, little is known of the evolutionary robustness of the mechanisms underlying this event. We recorded the first embryonic division of 42 species of nematodes closely related to *Caenorhabditis elegans*, which is an excellent model system to study the biophysical properties of asymmetric spindle positioning. Our recordings, corresponding to 128 strains from 27 *Caenorhabditis* and 15 non-*Caenorhabditis* species (accessible at http://www.ens-lyon.fr/LBMC/NematodeCell/videos/), constitute a powerful collection of subcellular phenotypes to study the evolution of various cellular processes across species. In the present work, we analyzed our collection to the study of asymmetric spindle positioning. Although all the strains underwent an asymmetric first cell division, they exhibited large intra- and inter-species variations in the degree of cell asymmetry and in several parameters controlling spindle movement, including spindle oscillation, elongation, and displacement. Notably, these parameters changed frequently during evolution with no apparent directionality in the species phylogeny, with the exception of spindle transverse oscillations, which were an evolutionary innovation at the base of the *Caenorhabditis* genus. These changes were also unrelated to evolutionary variations in embryo size. Importantly, spindle elongation, displacement, and oscillation each evolved independently. This finding contrasts starkly with expectations based on *C*. *elegans* studies and reveals previously unrecognized evolutionary changes in spindle mechanics. Collectively, these data demonstrate that, while the essential process of asymmetric cell division has been conserved over the course of nematode evolution, the underlying spindle movement parameters can combine in various ways. Like other developmental processes, asymmetric cell division is subject to system drift.

## Author summary

The cell is a level of biological organization that has been poorly explored from an evolutionary perspective because basic cell functions (e.g., cell division) show remarkable conservation across phyla. Thus, an essential question remains: to what extent can cellular mechanisms evolve without altering the basic function they sustain? We chose the asymmetric embryonic division of nematodes as a system to address this question. The first embryonic division of most nematode species gives rise to two daughter cells of unequal size and fate due to the asymmetric positioning of the mitotic spindle. This initial event is crucial to embryogenesis and has been extensively studied in the nematode species *Caenorhabditis elegans*. In this study, we recorded the first embryonic division of 42 nematode species, corresponding to 128 different strains and approximately 1,320 embryos, and characterized spindle movements. We found that significantly different combinations of spindle movements ultimately led to an asymmetric positioning of the spindle and observed that even between virtually identical phenotypes, spindle mechanics differed. We thus conclude that asymmetric cell division is an essential cellular function that is maintained over the course of evolution while the underlying mechanism that sustains it—asymmetric spindle positioning—changes rapidly. In addition, our collection of subcellular phenotypes constitutes a powerful resource to investigate other evolutional aspects of cellular processes.

## Introduction

During animal cell division, the cleavage plane is usually dictated by the position of the mitotic spindle at the end of mitosis; thus, the final spindle position influences the size of the two daughter cells [1]. In polarized cells, the spindle orientation with respect to the polarity axis determines the cellular content, and thus the fate, of the daughter cells [2]. Because spindle positioning plays an essential role in stem cell renewal and cell fate decisions during development, studies of oriented cell division in a variety of model systems have intensified over the past two decades [3,4]. These studies have revealed that, in most systems, oriented cell division relies on common principles and conserved molecules, particularly the establishment of cell polarity through asymmetric localization of the conserved PAR proteins and of conserved force generators (i.e., cortically anchored dynein complexes) that pull on astral microtubules in response to PAR polarity [3,4]. While these processes have been well studied in divergent organisms, such as worms and mice, and various cell types, such as embryonic cells and neuronal stem cells [3,4], much less is known about the evolutionary robustness of the mechanisms underlying oriented cell division. To explore the evolution of asymmetric mitotic spindle positioning, we analyzed the process in the same cell type in a panel of closely related species with comparable cellular architecture, namely the first embryonic cell division in 42 *Caenorhabditis* and non-*Caenorhabditis* nematode species, corresponding to 128 strains.

The first embryonic division of *Caenorhabditis elegans* is typically asymmetric, yielding two daughter cells that differ in both size and fate, and it has been a particularly important model for deciphering the mechanisms of asymmetric spindle positioning in animal cells [5]. *C*. *elegans* has many advantages as an experimental system for these studies [6–8]. In particular, the large size, transparency, and rapid cell cycle of the one-cell-stage *C*. *elegans* embryo and the stereotypical spindle movements allow the cellular events to be visualized with excellent spatiotemporal resolution using differential interference contrast (DIC)/Nomarski microscopy.

In the one-cell embryo of *C*. *elegans*, the antero/posterior axis is established at fertilization by the asymmetric partitioning of PAR polarity proteins on either side of the cell [9,10]. In response to this polarity, the pronuclei and spindle undergo stereotypical movements [5]. First, the male and female pronuclei meet at the posterior side of the cell and form a complex with the paternally provided centrosomes (the nuclei centrosome complex [NCC]). Next, the NCC migrates anteriorly and aligns along the antero/posterior axis. Metaphase spindle formation therefore occurs centrally. This is caused by a slight enrichment of the Gα/GPR/LIN-5/dynein protein complex on the anterior cortex, which generates unbalanced forces that pull on astral microtubules toward the anterior side of the cell [11,12], combined with microtubule length-dependent pulling forces that are generated by dynein motors acting along the microtubule lattice [13,14]. At the onset of anaphase, the Gα/GPR/LIN-5/dynein complex becomes modestly enriched on the posterior cortex and generates higher net forces on that side of the cell [11,15–17]. Consequently, the spindle is displaced posteriorly as it elongates. During spindle displacement, cortical pulling forces also induce regular out-of-phase spindle oscillations on the transverse axis of the cell. Here too, the asymmetric forces in the cell bias the oscillation amplitude such that it is always higher for the posterior than the anterior centrosome [6,18,19]. Complete inactivation of the dynein complex abolishes spindle displacement and spindle rocking, whereas incomplete activation affects spindle oscillations but preserves the asymmetric spindle positioning, suggesting that oscillations emerge above a threshold of active forces [19]. Physical modeling and simulation of oscillations have been proposed, in which cortical force generators would pull from the upper and lower cortex and a slight displacement of the centrosome toward the upper cortex, for instance, would be amplified because pulling forces increase as the centrosome nears the cortex [19–21]. Hypothetically, this could result from a decrease in load per motor as the distance to the centrosome decreases [19]. A restoring force would then return the centrosome to the center of the cell, possibly generated by astral microtubules pushing on the cortex as they polymerize [19] or by buckling of the microtubules, extending them laterally to the oscillation axis [21]. Importantly, although spindle oscillations are not essential in achieving asymmetric cell division, they reflect the mechanical properties of the spindle, and oscillations behavior have been instrumental in understanding the biophysical and molecular basis of asymmetric cell division in *C*. *elegans* embryos [5].

*C*. *elegans* is a member of the family of Rhabditidae nematodes, for which a phylogeny is well established [22]. All Rhabditidae species so far examined undergo a first embryonic division asymmetric in both size and fate of the daughter cells [23–25]. Intriguingly, earlier work on species that display the same spatiotemporal resolution of cellular events by DIC microscopy as *C*. *elegans* had observed variations in spindle movements during asymmetric embryonic cell division [25]. To further explore spindle mechanics on both small and large evolutionary distances, we precisely quantified spindle movements during the first embryonic division in 42 nematode species, from the same genus as *C*. *elegans* and from 10 other genera, and analyzed several strains per species.

Our study uncovered the fact that, although all species underwent an asymmetric cell division, the degree of cell asymmetry and more particularly spindle movements varied considerably within and between species. We found independent variations in spindle elongation, displacement, and oscillations that were unexpected based on our understanding of *C*. *elegans*. Moreover, the variations in spindle movements were unrelated to variations in cell size and did not predict the degree of cell asymmetry. Lastly, we found that spindle movements generally changed many times during evolution, with no obvious trends in the phylogeny. The exception was spindle oscillations, which constituted an evolutionary innovation at the base of the *Caenorhabditis* genus. Thus, although the developmental program among nematodes is constrained by asymmetric cell division, the underpinning of this asymmetry, i.e., the positioning of the spindle, has undergone rapid and frequent change. Finally, our study provides a powerful resource, with a detailed quantitative description of subcellular traits, that could be reused to investigate other evolutionary aspects of cell and developmental processes.

## Results

### Cellular traits of the first embryonic division show large intra- and inter-species variations

**Fig 1:**
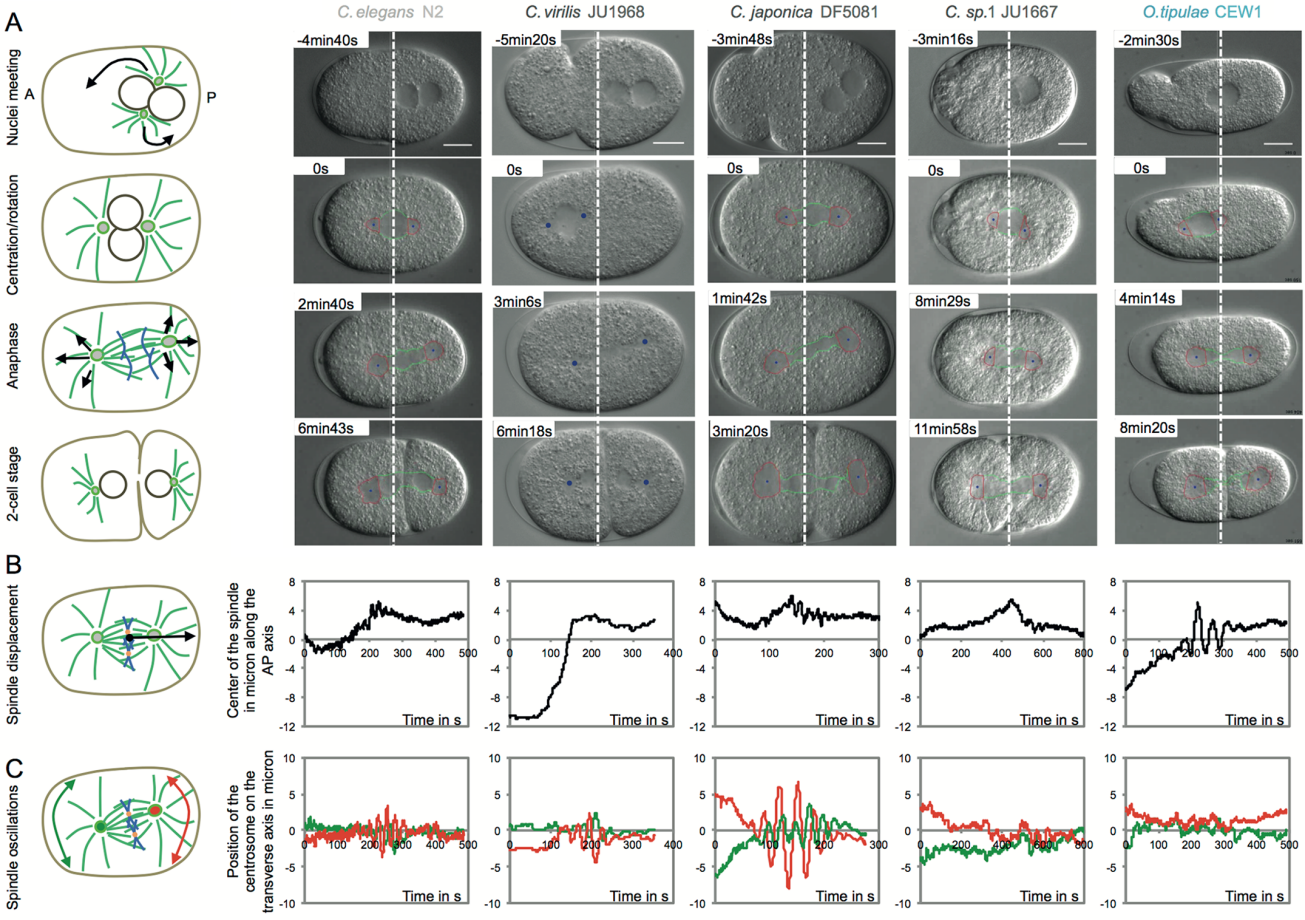
Patterns of spindle movements among various nematode species. (A) (Left) Schematic representations of an embryo from the meeting of the pronuclei to the first cell division. Microtubules are shown in green and chromosomes in blue. Mechanical forces pulling on astral microtubules are responsible for nuclei and spindle movements and are shown as black arrows. (Right) DIC still images of embryos (at stages corresponding to the schematics) from four *Caenorhabditis* species (*C*. *elegans* and three species from the non-*Elegans* group) and *Oscheius tipulae* from another genera. Anterior and posterior sides are left and right, respectively. The white dotted line represents the middle of the cell. Centrosomes (center in blue and contour in red) were detected by an automated program for all these species except for *C*. *virilis*, where the centrosomes have been tracked manually (shown as blue dots). Scale bar: 10 μm. (B, C) Time course of displacement of the spindle center along the antero/posterior axis of the cell, relative to the center of the cell (B) and transverse oscillations of anterior (green) and posterior (red) centrosomes relative to the cell equator (C) for the five species shown above. *y* and *x* axes are measured in μm and s, respectively. T = 0s corresponds to the onset of nuclear envelope breakdown. Corresponding numerical values are provided in S3 Table. DIC, differential interference contrast.

We compared the laboratory strain *C*. *elegans* N2 with 27 closely related species in the *Caenorhabditis* genus, 10 of which were in the “*Elegans* group” and 17 of which were in the “non-*Elegans* group,” as defined previously [26]. We also selected 15 species in 10 genera more distantly related to *C*. *elegans*, which we refer to as “non-*Caenorhabditis* species” [22] (S1 Table). Before comparing spindle positioning during the first embryonic division, we examined the general organization of the spindle in a subset of *Caenorhabditis* and non-*Caenorhabditis* species. Using tubulin immunostaining to visualize microtubules, we found that the anaphase spindle presented as a long array of microtubules between the centrosomes, with a strong density of astral microtubules associated with each centrosome in all species examined, similar to the structure observed in *C*. *elegans* (S1 Fig). Thus, we could compare spindle positioning between species that show a similar overall spindle architecture. Next, we analyzed spindle movements during asymmetric cell division by performing time-lapse DIC videomicroscopy of the first embryonic cell division in 128 nematode strains from the 42 species (listed in S1 Table). From these recordings, we extracted traits associated with cell size and shape and automatically detected the position of the centrosomes over time to quantify the spindle size and movements (Fig 1, S2 and S3 Tables, and Materials and methods) and compare them between strains and species (S4 Table).

We first examined cell size, shape, and asymmetry. In *C*. *elegans* N2, the one-cell embryo has an oblong shape and divides asymmetrically (Fig 1). As previously described [27], we observed large variations in embryo length and width among species (S4 Table and S2A Fig), resulting in marked differences in the cell aspect ratio (cell length/width) even though the overall oblong shape was maintained (S3A and S3B Fig, S4 Table). In the *Caenorhabditis* genus, the cell aspect ratio ranged from 1.4 to 1.8, while this ratio was > 2 for some non-*Caenorhabditis* species, such as *Pristionchus maupasi* RS0144 and the tiny embryo of *Diploscapter* sp. JU359. We next quantified the asymmetry of the first cell division by measuring the absolute position of the division plane in microns or by measuring the relative asymmetry between daughter cells (S2B and S3C Figs, S4 Table, and Materials and methods). We found variations in both parameters. Interestingly, although the relative asymmetry of division was variable, it was globally constrained: it ranged from 1.11 in mean value for *Pristionchus pacificus* PS312 to 1.46 for species showing the most pronounced asymmetry, such as *C*. *remanei* MY204 and *C*. *brenneri* JU1817 (the asymmetry for *C*. *elegans* N2 was 1.29) (S3C Fig). Thus, while all 42 species had oblong embryos that divided asymmetrically, the length, width, and aspect ratio of the embryo and the degree of daughter cell asymmetry varied between strains and species.

**Fig 2:**
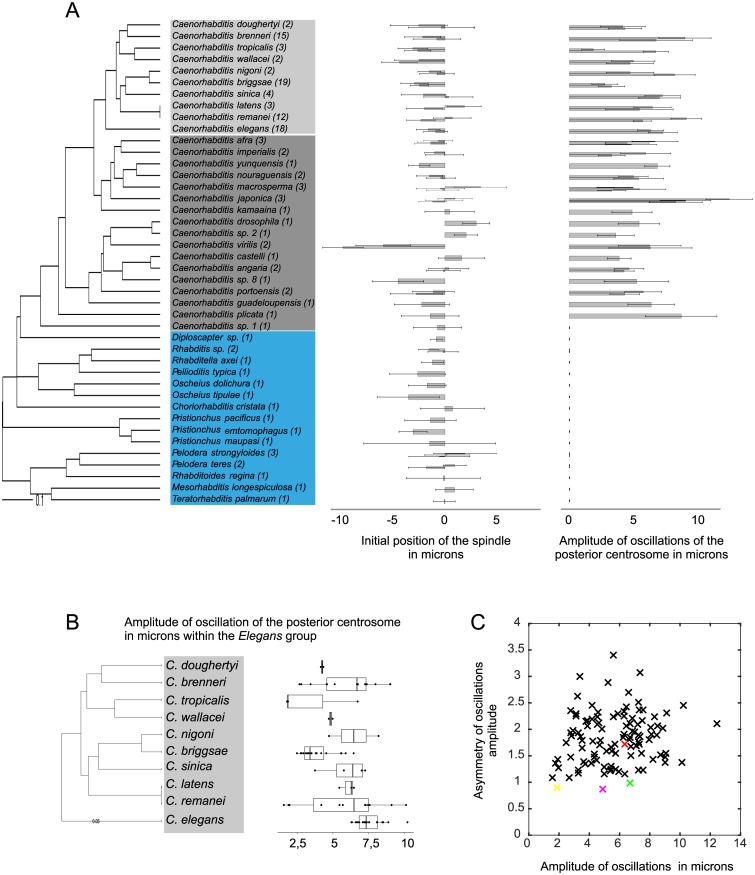
Intra- and inter-species variations in cellular traits. (A) (Left) Phylogeny of the species used in this study. Numbers in parentheses are strains per species. Within the *Caenorhabditis* genus, species from the *Elegans* group are shown in light gray, from the non-*Elegans* group in dark gray. Other genera are shown in blue. (Right) Initial position of the spindle relative to the cell center and amplitude of oscillations of the posterior centrosome (both in μm). Bars represent the mean ± SD of all strains per species except for the *Elegans* group, for which two strains per species were chosen at random. Underlying numerical values are shown in S5 Table. (B) Box and whisker plot of the amplitude of oscillations (μm) of the posterior centrosome for all species of the *Elegans* group. Each dot corresponds to one strain, and the box and whiskers represent the median, quartiles, and 1.5 interquartile range. Underlying numerical values are provided in S4 Table. (C) Asymmetry of the maximum oscillation amplitudes (ratio of the posterior centrosome maximum amplitude over the anterior centrosome maximum amplitude) relative to that of the posterior centrosome (μm). Each cross represents a strain, with *C*. *elegans* N2 in red, *C*. *tropicalis* JU1428 in yellow, *C*. *kamaaina* QG122 in magenta, and *C*. *brenneri* VX0044 in green. The latter three are examples of species showing equal oscillation amplitudes for the anterior and posterior centrosomes. Underlying numerical values are provided in S4 Table.

Next, we analyzed spindle displacement during anaphase. In *C*. *elegans* N2, the spindle is centrally located at the onset of mitosis and later becomes displaced toward the posterior of the cell because of unbalanced cortical pulling forces that are differentially regulated during the cell cycle (Fig 1A). We found that many strains showed nuclear off-centering posteriorly or anteriorly (S4 Table, Figs 1B and 2A). Thus, unlike *C*. *elegans*, the remaining species did not uniformly show centering of the nuclei at the onset of mitosis, despite having similar cellular shapes. Moreover, while all species showed posteriorly positioned spindles at the end of mitosis, the final position varied, resulting in large quantitative differences in spindle displacement during mitosis (S4 Table and S2C Fig). For instance, in *C*. *japonica* DF5081 (Fig 1) and *C*. sp. 2 DF5070, the pronuclei did not center during prophase; thus, since the spindle was already located posteriorly at the onset of anaphase, the total distance traveled during anaphase was particularly short (1.67 mean ± 1.16 SD μm and 0.84 ± 1.41 μm, respectively). *C*. *virilis* JU1968 illustrates the other extreme (Figs 1 and 2A). In this case, meeting of the pronuclei was followed by extensive migration anteriorly during prophase, spindle formation, and then spindle migration over 12.11 ± 2.2 μm during anaphase to reach its final position. These results suggested that the force balance responsible for nuclear and spindle positioning had changed between strains and species.

Spindle elongation in *C*. *elegans* N2 during anaphase is mediated by cortical forces pulling on astral microtubules on each side of the spindle. We found large intra- and inter-species variations in the initial spindle size and its final size at the end of mitosis, consistent with previous observations [27]. Interestingly, we also identified extensive variation in the rate of spindle elongation (S4 Table). While the increase in spindle length during mitosis was 1.92-fold in mean in *C*. *elegans* N2, it ranged from 1.82-fold (*C*. *sinica* JU727) to 2.79-fold (*C*. *nigoni* JU1325) in the *Elegans* group of species, from 1.64-fold (*C*. *japonica* VX0130) to 2.78-fold (*C*. *afra* JU1286) in the non-*Elegans* group, and from 1.42-fold (*Diploscapter* sp. JU359) to 2.34-fold (*Pristionchus entomophagus* RS0144) in non-*Caenorhabditis* species (S2D Fig). This result suggested that the mechanics of spindle elongation differed between strains and species.

Finally, we assessed spindle oscillations. Similar to the mechanism of spindle elongation, cortical forces pulling on astral microtubules during anaphase also trigger transverse oscillations of the centrosomes in *C*. *elegans* N2. The centrosomes oscillate out of phase, with the asymmetry of pulling forces causing more pronounced oscillations of the posterior centrosome compared with the anterior centrosome (Fig 1). We found that all *Caenorhabditis* species except *C*. sp. 1 displayed out of phase transverse oscillations of both centrosomes, similar to the movements observed in *C*. *elegans* (Fig 1). However, the amplitude of the centrosome oscillations showed both inter- and intra-species variations (Fig 2A–2C, S4 Table, and Materials and methods). For instance, the maximum amplitude of the posterior centrosome oscillations was 6.32 ± 1.02 μm (mean ± SD) for *C*. *elegans* N2, but it was much smaller (1.56 ± 0.45 μm) for *C*. *remanei* VX003 and much larger (10.11 ± 2.41 μm) for *C*. *remanei* PB219. It was also very high for all the *C*. *japonica* strains (from 8.29 ± 1.81 μm to 12.45 ± 1.81 μm) (Fig 1). The frequency of oscillations also varied between species, ranging from 29 mHz (*C*. *plicata* SM355) to 64 mHz (*C*. *briggsae* HK104), as did the duration of oscillations, with mean values ranging from 64 s (*C*. *remanei* VX0003) to 369 s (*C*. *doughertyi* JU1333) (S4 Table). Nevertheless, the majority of species showed larger oscillation amplitudes for the posterior centrosome than for the anterior centrosome, consistent with the systematic posterior displacement of the spindle (Fig 2C). Of note, the degree of asymmetry between the anterior and posterior oscillations was highly variable (Fig 2C). For example, *C*. *elegans* showed a 1.72-fold larger posterior oscillation amplitude than anterior oscillation amplitude, whereas this asymmetry was 3.4-fold for *C*. *briggsae* QR24 (S4 Table). Interestingly, a few strains showed virtually identical oscillation amplitudes for both centrosomes, independently of the amplitude size. In *C*. *tropicalis* JU1428, both centrosomes oscillated with amplitudes of 2 μm, while in *C*. *brenneri* VX0044, the amplitudes were 6.7 μm (Fig 2C). Such large variations in the extent (i.e., duration, frequency, amplitude) of spindle oscillations and in the degree of asymmetry between the anterior and posterior oscillations are indicative of frequent evolutionary changes in the balance of forces acting on the spindle. This result also suggests that the asymmetry of spindle oscillations does not reflect the asymmetry of spindle positioning within *Caenorhabditis* species. Interestingly, we found that *C*. sp. 1, the most basal species of the *Caenorhabditis* genus, and all non-*Caenorhabditis* species did not exhibit stereotypical spindle oscillations, although the centrosomes did show some transverse movements (Figs 1A, 1C and 2A). Collectively, these data indicate that spindle oscillations are an evolutionary innovation that appeared at the base of the *Caenorhabditis* and that spindle mechanics have changed markedly between *Caenorhabditis* and the other genera examined.

Given these intra-species phenotypic variations, we asked whether inter-species differences were apparent when the mean species trait values were compared. Indeed, using a nonparametric Kruskal—Wallis comparison, we found a significant difference in parameter distribution between species for 20 out of 22 traits (with a False Discovery Rate [FDR] of 0.001; S6 Table). Thus, we concluded that most of the measured morphological traits showed significant inter-species differences, despite intra-species variation. Taken together, these results revealed that evolutionary changes have taken place in the size and shape of the embryo, the degree of cell asymmetry, and the extent of spindle displacement, elongation, and transverse oscillations in nematodes, despite their similar spindle architecture and conserved asymmetric cell division.

### Variations in spindle movements are not explained by evolutionary changes in cell size

**Fig 3:**
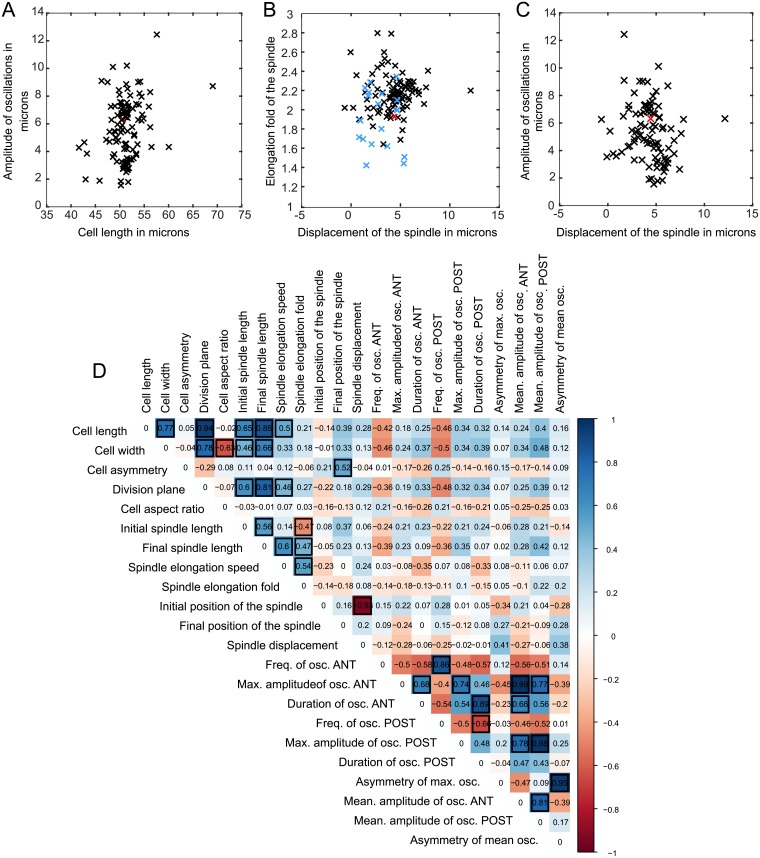
Covariation of traits. (A-C) Individual data plots showing absence of covariation of the indicated traits among the strains (*Caenorhabditis* genus in black, other genera in blue, *C*. *elegans* N2 in red). Underlying numerical values are shown in S4 Table. (D) Covariation between pairs of parameters, estimated by phylogenetic correlations. Color scale shows similarity scores and black outlined squares indicate significant correlations after correction for multiple testing (1,000 random samples of one strain per species, adjusted *p*-value < 0.05). For parameters associated with spindle oscillations, only strains of the *Caenorhabditis* genus undergoing spindle oscillations were included. Corresponding *p*-values are provided in S7 Table. ANT, anterior; max., maximum; osc., oscillation; POST, posterior.

We next asked whether intra-species variations in cellular asymmetry and spindle behavior could be explained by basic scaling arguments arising from changes in cell size over the course of nematode evolution. Traits associated with spindle size were previously shown to be strongly correlated with cell length and width across nematode species, suggesting that such scaling may be a conserved property during embryo development across the phylogeny [27]. To determine whether a relationship exists between cell size, cell asymmetry, and spindle movements, we measured pairwise correlations between the traits in the 128 nematode strains examined, taking into account the phylogenetic relationship between species (Fig 3A and 3D, S3 Fig, and Materials and methods). We confirmed that the initial and final spindle sizes, as well as the speed of spindle elongation, correlated with cell length (R = 0.65, 0.86, and 0.50, respectively; all greater than 0 with a FDR of 0.05). As expected also, the position of the division plane correlated strongly with cell length (R = 0.94). However, the relative asymmetry of division, the fold-change in length (elongation fold) of the spindle, the position of the spindle at the onset and end of mitosis, and traits associated with spindle oscillation (amplitude, frequency, and duration) did not correlate with cell length, width, or aspect ratio (Fig 3D). Thus, evolutionary changes in cell length can explain the pattern of variation in spindle size [27], but not in traits associated with spindle movements or in relative cell asymmetry.

### Variations in spindle movements and cellular asymmetry are not due to differences in tuning of the conserved force-generating machinery

We next considered that the observed inter-species differences in spindle movement and cellular asymmetry could result from altered tuning of the conserved spindle-pulling machinery. In *C*. *elegans* N2, modulation of this machinery by genetic manipulation of the cortical force generators leads to simultaneous changes in spindle elongation, in displacement, and in rocking of the spindle. For example, up-regulation of GPR expression leads to NCC overcentration to the anterior side of the cell and to exaggerated rocking of the centrosomes during anaphase [28,29]. In contrast, GPR down-regulation reduces anaphase spindle elongation, displacement, and rocking [15–17,19]. Based on these observations, we expected that evolutionary changes in the strength of pulling forces would result in covariation of all traits associated with spindle movements. However, we found many strains with an unexpected combination of spindle movements. In one example, overcentration of the NCC was followed by markedly reduced amplitude of spindle oscillations in *C*. *brenneri* JU1886, while reduced spindle elongation and concomitantly increased spindle oscillations were found in *C*. *japonica* (Figs 1, 3B and 3C). Therefore, we systematically analyzed the covariation of cell asymmetry and spindle elongation, displacement, and oscillations in the 128 different nematode strains by measuring pairwise correlations between the traits, taking phylogeny into account (Fig 3D). This analysis revealed several important relationships. First, among the species that displayed spindle oscillations (i.e., all *Caenorhabditis* species except *C*. sp. 1), the frequency, amplitude, and duration of oscillations of the anterior centrosome always covaried with those of the posterior centrosome (Fig 3D, R = 0.86, 0.74, and 0.89, respectively; FDR < 0.05). However, no correlation was found between the frequency and the amplitude of oscillations for a given centrosome. Thus, as previously proposed for *C*. *elegans* [19,20], our results revealed that while the amplitude and frequency of spindle oscillations are controlled independently, the anterior and posterior centrosome oscillations are mechanistically linked. Taking all species into account, we also found a strong correlation between the initial position of the spindle and its overall displacement, as expected (R = −0.93, FDR < 0.05), with anteriorly positioned spindles traveling greater distances than initially posteriorly positioned spindles. However, spindle displacement did not correlate with the extent of spindle elongation. For species displaying spindle oscillations, we also found that neither spindle displacement nor elongation correlated with oscillation frequency/amplitude/duration. This absence of evolutionary covariation between traits associated with spindle elongation, displacement, and transverse oscillations strongly suggests that spindle movements are not controlled by a single mechanism that is tuned slightly differently in each species, but rather by distinct spindle elongation, displacement, and rocking mechanisms that have changed independently over the course of nematode evolution. Lastly, although we found, as expected, a correlation between the final position of the spindle and the relative cell size asymmetry (R = 0.52, FDR < 0.05), no correlation between other spindle traits and the degree of cell asymmetry was found. From this, we concluded that spindle movements, i.e., oscillations, elongation, and displacement, did not predict the degree of cellular asymmetry in nematodes.

### The asymmetry of division is controlled by strain-specific combinations of spindle movements

**Fig 4:**
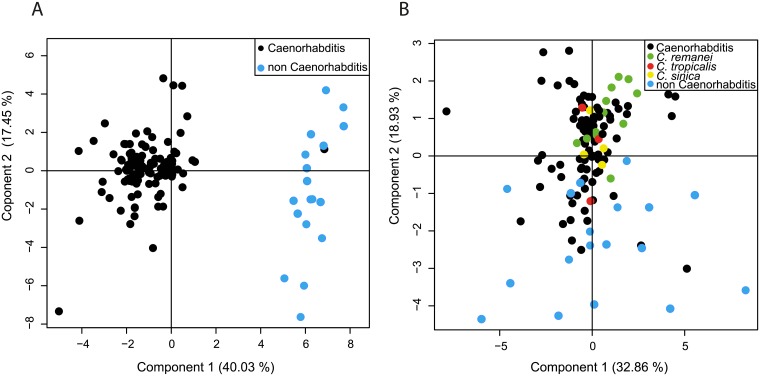
Repartition of strains on the phenotypic space. (A, B) PCA for all strains. Underlying numerical values are provided in S8 Table. (A) All parameters are taken into account. *Caenorhabditis* species in black, non-*Caenorhabditis* species in blue. (B) All parameters except those associated with spindle oscillations are taken into account. Non-*Caenorhabditis* species are in blue; *Caenorhabditis* species in black. *C*. *remanei* in green, *C*. *tropicalis* in red, and *C*. *sinica* in yellow. PCA, principal component analysis.

Our results suggest that the balance of forces acting on the spindle to achieve asymmetric positioning exhibited inter- and intra-species mechanistic differences. To probe this further, we asked whether we had a continuum of subcellular phenotypic features among strains or whether discrete classes of strains with distinct subcellular phenotypes existed. In the latter case, such classes could correspond to strains that are phylogenetically close or that are, for instance, living under the same climate. In order to analyze the position of strains in such a phenotypic space, we performed principal component analysis (PCA) of all strains, taking into account all the measured parameters, as described for yeast strains in [30] (see Materials and methods). We found that the first PCA component explained 40.03% of the total variance and clearly split the species into two groups based on the presence or absence of spindle oscillations (i.e., *Caenorhabditis* except *C*. sp. 1 and non-*Caenorhabditis*) (Fig 4A). As expected, *C*. sp. 1, which does not display spindle oscillations and diverges basally in the *Caenorhabditis* genus, grouped with the non-*Caenorhabditis* species in this analysis.

We next performed the PCA after excluding the parameters associated with spindle oscillations (frequency, amplitude, and duration). Notably, strains belonging to the same genus were no longer grouped on either the first (32.86%) or second (18.93%) PCA axis (Fig 4B). Moreover, in some instances, the difference between strains of the same species was as large as that between distinct species, as illustrated by *C*. *sinica*, *C*. *tropicalis*, and *C*. *remanei*, for example.

We also asked whether different combinations of parameters were shared by strains isolated from geographical regions with the same climate (e.g., temperate, tropical, monsoon, oceanic). However, no significant relationships were detected, suggesting that spindle movement differences had emerged independently of climate (S4 Fig).

Thus, with the exception of the presence or absence of spindle oscillations, no patterns of cell size and shape or spindle size and motion emerged that could clearly discriminate between nematode strains or species. Combined with the absence of covariation of traits, this result suggests that strains are characterized by a unique combination of spindle movements during asymmetric spindle positioning (or specific phenotype).

### Cellular traits of the one-cell embryo have changed repeatedly during nematode evolution

**Fig 5:**
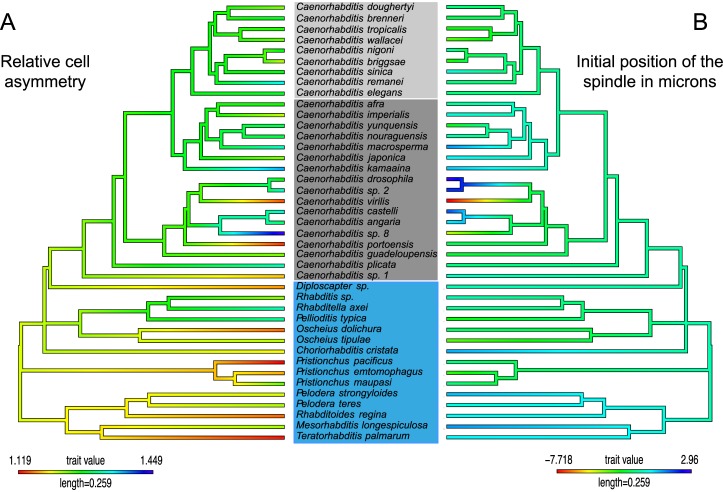
Map of individual parameters on the phylogeny of species. Phylogenetic tree colored according to the mean values per species of relative cell size asymmetry (A) and the initial position of the spindle in μm relative to the cell center (B).

Because we did not identify common groups of strains or species that shared combinations of spindle movement parameters, we next asked whether some of the individual parameters measured showed a specific trend of changes along the phylogeny. We reasoned that certain parameters, such as spindle length or cell asymmetry, might be shared by species monophyletic subgroups, similar to the restriction of spindle oscillations to the *Caenorhabditis* genus. We first mapped the mean value of each character per species on the phylogeny and found no obvious trend, except for the absence of oscillations in non-*Caenorhabditis* species (Fig 5, S5 Fig, Materials and methods). This pattern is compatible with a Brownian motion model, where the divergence between two phenotypes is proportional to the time elapsed since their common ancestor, similar to a random walk. Alternatively, it could reflect the existence of an optimum value for a parameter, around which stabilizing selection allows small changes between species (Ornstein–Uhlenbeck model). We compared the two models for all traits and found that the Ornstrein–Uhlenbeck model was not preferred over the simple Brownian motion model, except for traits related to spindle oscillations (computation using geiger package [31]; S9 Table). However, within the *Caenorhabditis* genus, the Ornstrein–Uhlenbeck model was not preferred over the Brownian motion model for traits related to spindle oscillations. This result showed that once oscillations emerged in the *Caenorhabditis* genus, oscillation traits changed many times within the genus. All the other subcellular traits also showed frequent alterations with no specific directionality over the course of nematode evolution, despite having a common constraint on the final phenotype, i.e., the asymmetry of division.

## Discussion

### A powerful collection of subcellular phenotypes

Asymmetric cell division is an essential biological function and has been examined extensively in different cell types and model organisms [3,4]. To explore the evolution of this crucial event, we compared a number of traits associated with asymmetric spindle positioning in homologous cell types from species of a common origin. We selected nematodes of the Rhabditidae family, in part because the embryos of all species undergo a first asymmetric cell division and they display similar cellular and spindle architecture, allowing us to visualize cellular events with the same spatiotemporal resolution as the comparator model species *C*. *elegans*, which has been studied extensively [5].

Using DIC microscopy, we recorded the first embryonic division of 128 strains from 42 nematode species of the Rhabditidae family, thus establishing an extensive collection of subcellular phenotypes. Although our work has focused mainly on the analysis of spindle size [27] and motion (this study) to explore the evolution of spindle mechanics, our data can serve as a resource to address many other types of questions. First, from our measurements, specific species and strains can be selected for further functional studies, for instance, to study the evolution of embryo size, the mechanisms of nuclei centering, etc. The measured traits could also be correlated with other types of measurements on body size (to study allometry), later embryonic cell divisions (to study the developmental variability of early versus late developmental stages), etc. Lastly, our recordings (accessible at http://www.ens-lyon.fr/LBMC/NematodeCell/videos/) can also be used to explore the evolution of other important cellular features for which we have also observed large interspecies variations without quantification, such as nuclear size, membrane contractility, or cell cycle duration.

### Unexpected intra- and inter-species variations in subcellular traits

All of the nematode species selected undergo a first asymmetric embryonic division. Unexpectedly, we found that the degree of asymmetry between the two daughter cells varied. We also uncovered large intra- and inter-species variations in spindle movements. Despite these variations, we found a significant difference in trait distribution between species. Interestingly, when we compared two self-fertilizing hermaphroditic species, *C*. *elegans* and *C*. *briggsae*, and two gonochoristic species, *C*. *remanei* and *C*. *brenneri*, for which we had analyzed more than 12 strains per species, we did not find a systematic increase of trait variation among strains of outbreeders compared to strains of selfers (S6 Table, pairwise F test). Indeed, for 18 quantitative traits out of 22, the distribution of the variance did not differ between the two groups. For neutrally evolving molecular markers, the theory predicts that polymorphism should be lower in selfers because the effective population size is lower [32]. In agreement with the theory, the outbreeder *C*. *remanei* has been shown to harbor a large genetic diversity, while *C*. *brenneri* is the most hyperdiverse species described to date [33,34]. In the case of morphological phenotypes, the expectations are much less clear [33,35], and our results have shown that outbreeders do not systematically harbor a higher variance compared to selfers. Thus, in our dataset, variations in these subcellular traits do not directly reflect the species’ reproductive strategies. We also uncovered that the variations do not reflect a specific adaptation to the geographical and climatic origins of these species.

### Evolutionary changes in spindle mechanics during conserved asymmetric cell division

It is important to note that the differences in spindle motion, i.e., spindle displacement, elongation, or rocking, were also not due to evolutionary changes in embryo size or shape and were not correlated with the asymmetry of division. Thus, spindle movements per se could not predict the asymmetry of division. Moreover, we found no similarity between *C*. *elegans* and many other species with respect to the combination of spindle movements along the antero/posterior axis (i.e., displacement and elongation) or transverse axis (i.e., rocking) of the cell. This result strongly suggested that evolutionary changes in the balance of forces acting on the spindle, rather than a modulation of the same mechanics among species, was responsible for the different pattern of spindle motion observed. In this regard, we previously analyzed the pattern of spindle motion in *C*. *briggsae*, which is characterized by overcentration of the NCC during prophase, reduced spindle oscillations during anaphase, and the presence of a single *gpr* gene in the genome, in contrast to two *gpr* copies in the *C*. *elegans* genome [36]. We demonstrated that modulation of GPR levels in *C*. *briggsae*, and consequently, the strength of cortical pulling forces, was indeed not sufficient to mimic the phenotype of *C*. *elegans* embryos and vice versa [36]. With this new study, we further revealed that the mechanical forces controlling spindle displacement, elongation and transverse oscillations changed frequently and independently over the course of nematode evolution. Studies in *C*. *elegans* had shown that different spindle movements could be experimentally uncoupled. Physical destruction of centrosomes in *C*. *elegans* embryos, which abolishes the source of pulling forces, does not prevent spindle elongation [37]. Similarly, a slight diminution of components of the force generator complex in *C*. *elegans* embryos prevents transverse spindle oscillations but not spindle displacement [19]. We propose that this uncoupling allowed the mechanistic underpinnings of spindle elongation, displacement, and oscillation to change many times over the course of nematode evolution because various combinations of these mechanisms appear equally able to support asymmetric spindle positioning.

### Evidence of cellular system drift

Some phenotypes remain unchanged over long periods of time. Such evolutionary stasis can result from two evolutionary scenarios. In one, strong constraints due to physical properties of the system or genetic architecture may prevent variations in the underlying mechanisms, leading to constancy in the output phenotype [38]. In the second scenario, the underlying processes may change, but the output phenotype remains invariant. The latter case is referred to as cryptic evolution or system drift [39]. Thus far, system drift has only been explored in a few examples of conserved developmental processes [40–43]. Basic cellular processes, such as cell division or transport of molecules, show remarkable conservation across species, leading to the assumption that they share common underlying mechanisms. Consequently, little attention has been paid to the evolution of cellular mechanisms [44], and a number of critical questions remain. For example, how far can cellular mechanisms diverge without affecting the function they sustain? Which parameters supporting a given function are inherently flexible and allow change? Here, we addressed these questions in the context of asymmetric positioning of the spindle and discovered that different spindle movement parameters can combine in various ways, but they ultimately result in the same output phenotype, i.e., an asymmetric cell division. These data support the notion that, like developmental processes, basic cellular functions are subject to system drift.

The existence of system drift does not, however, imply that neutral genetic drift rather than selection is responsible for the observed cryptic variation. We found that variations in all traits were compatible with a model of Brownian motion evolution, in which the phenotypic divergence between a pair of species is proportional to the time spent since their last common ancestor. This Brownian motion model classically reflects phenotypic evolution by neutral genetic drift, but it can also reflect the existence of multiple optimums along the phylogeny. The expected variation for traits evolving solely by neutral drift can be deduced from an estimation of the amount of genetic variance generated by spontaneous mutation in a single generation and the known effective size of the population [45,46]. By a thorough analysis of the similar parameters than those quantified in this study in the species *C*. *elegans*, whose population size is known [47], Farhadifar et al. had previously shown that the level of trait variation was lower than expected from a model of neutral drift [48]. However, our data do not allow us to explain the origin—neutral or not—of cellular system drift in spindle mechanics across species, because for the majority of those species, we ignore the effective population size as well as the genetic variance generated by spontaneous mutations.

Importantly, the existence of system drift implies that a mechanism essential to one species might be dispensable to another in which other mechanisms may perform the same function. Our study has confirmed that the commonly accepted mechanism of spindle positioning, based on extensive analyses of *C*. *elegans*, cannot be simply transposed to other species. This observation reinforces the need to extend our exploration of basic cellular processes—despite their apparent conservation—beyond a few model organisms to a panel of species.

### Exploring the cellular parameter combinations that support asymmetric spindle positioning

A number of scenarios could explain the observed inter-species variations in spindle rocking and displacement along the anteroposterior axis of the cell. The spindle of *C*. *kamaaina* embryos undergoes symmetric spindle oscillations while it is posteriorly displaced during anaphase. Differences in cytoplasmic viscosity or microtubule dynamics at the anterior and posterior poles of the cell could explain why both centrosomes oscillate similarly upon asymmetric cortical pulling forces. A concentration of cortical force generators at the tip of the cell rather than distributed on the half cortices could possibly explain why cortical force generators do not generate transverse spindle oscillations in non-*Caenorhabditis* species. Further exploration of the mechanistic basis of spindle motion in a subset of species will be required to identify the most flexible and evolvable parameters. The genomes of many Rhabditidae species have been or are currently being sequenced, which will facilitate the analysis of evolutionary changes in the molecular players in spindle dynamics. Genetic approaches could be envisaged for species that are amenable to functional analysis [49–51] and that display phenotypes resembling those of *C*. *elegans* mutants, as previously performed in comparisons of *C*. *briggsae* and *C*. *elegans* embryos [36]. For instance, *C*. *macrosperma* JU1853 shows reduced amplitude of spindle oscillations but greater spindle elongation compared with *C*. *elegans*. This phenotype is similar to that of *C*. *elegans* embryos lacking EFA-6, a protein that limits microtubule growth at the cortex [52]. Therefore, monitoring and manipulating microtubule dynamics in *C*. *macrosperma* could identify some of the molecular changes that underlie the differences in spindle mechanics between these species. In *C*. *elegans*, the LET-99 protein has a peculiar cortical localization in a posterior band domain, which creates asymmetry in the repartition of force generators contributing to NCC centration [53]. Divergence in LET-99 function and/or localization could explain the NCC off-centering observed in some species, such as *C*. *virilis*. In our species list, *C*.sp. 1 represents a transition between species that do or do not display spindle oscillations. It will therefore be interesting to determine whether the absence of oscillations results from different tuning of the conserved machinery responsible for transverse movements or whether new mechanisms and molecules evolved to allow the emergence of spindle oscillations within the *Caenorhabditis* genus. *C*. *afra* and *C*. *nigoni* show the greatest spindle elongation during anaphase and may thus have central spindles with unusual and/or unique properties. Lastly, exploration of intra-species variations using quantitative trait locus analysis could be another fruitful approach to identifying the genetic basis for the drift in spindle motion. Because spindle movements involve the complex interplay between a number of cellular parameters, exploring their evolution in silico using mathematical models will be an extremely powerful approach to understanding spindle motion.

## Materials and methods

### Strains and culture conditions

We chose species in the Rhabditidae family that display the same experimental advantages of *C*. *elegans*, particularly the ease with which spindle movements can be monitored by DIC microscopy, the ability to feed on *Escherichia coli* in laboratory conditions similar to those used for *C*. *elegans*, and the ability to be frozen like *C*. *elegans*. To analyze functional evolution, we chose species with well-established phylogenetic relationships [22]. For most species, we only analyzed 1 to 4 natural isolates because of limited strain availability from the collections. However, we were able to select ≥ 12 strains for *C*. *elegans*, *C*. *remanei*, *C*. *briggsae*, and *C*. *brenneri*. For all strains, 8 to 29 embryos were analyzed and recorded, totaling approximately 1,320 embryos. Movies can be found at http://www.ens-lyon.fr/LBMC/NematodeCell/videos/. All strains (listed in S1 Table) were maintained at 20 °C on Nematode Growth Media supplemented with *E*. *coli* OP50 as a food source, as described in [54]. Since the submission of our manuscript, *Rhabditis* sp. TMG33 (S1 Table) has been renamed *Auanema rhodensis* TMG33 (see [62]).

### Embryo recording

Gravid females were cut open in standard M9 medium to release early embryos, which were then mounted in M9 onto 2% agarose pads between a slide and coverslip, as described in [55]. The samples were imaged by DIC microscopy on a Zeiss Axioimager A1 microscope equipped with a 100× Plan-Apochromat NA 1.4 lens. Embryos were imaged at 23 °C from the meeting of the male and female pronuclei until the end of the first cell division. Images were acquired every 0.5 s using a digital camera (DX4-285FW, Kappa) and the corresponding time-lapse module. This allowed us to capture rapid movements such as spindle oscillations.

### Event tracking and quantification

To quantify subcellular traits (S2 Table) in all embryos, we collected measurements from the DIC recordings (S4 Table). From the still images, we manually extracted four parameters describing cell size and shape using ImageJ software (National Institutes of Health). The aspect ratio was calculated as the cell length/cell width. The position of the cytokinetic furrow was measured at the end of mitosis as the length of the posterior P1 cell. It represented the absolute value of cell asymmetry. We also extracted the relative cell size asymmetry by calculating the ratio of the length of the anterior cell AB to the length of the posterior cell P1 at division.

Using a custom-designed automated tracking program [56,57] or using the “Manual tracking” plugin of Image J, we extracted the position of the posterior and anterior centrosomes over time, starting at the onset of mitosis, when centrosomes are clearly detectable by DIC microscopy, until the end of the movie. From this, 17 parameters associated with spindle size and spindle motion were obtained (S2 Table). The initial position of the nuclei before mitosis relative to the cell center was determined by measuring the distance between the most anterior position of nuclei and the center of the cell. This provided a measure of the centration process (negative values correspond to an overshooting of the nuclei in the anterior part of the cell). We excluded embryos for which recording had started after the rotation process since it was not possible to identify the most anterior position of the centrosome. The position of the centrosomes was measured at the end of mitosis, and the position of the center of the spindle was determined to evaluate the final position of the spindle relative to the center of the cell. The overall displacement of the spindle was calculated by subtracting the final position from the initial position of the spindle. Plots of spindle length over time were used to manually determine the initial size (first plateau) and the final size (the final plateau) of the spindle and the duration of spindle elongation. We determined the transverse movements of the centrosome to measure spindle oscillations relative to the center of the cell. The peaks and valleys of each oscillation were detected automatically from the oscillation curves generated by the program, from which we measured the frequency (mHz) and amplitude (μm) of each oscillation and the total duration (s) of oscillations (Fig 1). As the amplitude of oscillations vary over time, as previously shown for *C*. *elegans* [19], we used two different measurements to quantify the amplitude of oscillations: the maximum amplitude of oscillations and the mean of all successive oscillations for each centrosome. We also extracted the mean frequency of oscillations. Finally, oscillation asymmetry was calculated as the maximum (or mean) amplitude of the oscillation of the posterior pole divided by that of the anterior pole (S4 Table).

### Immunofluorescence microscopy

Embryos were processed for staining by fixing in methanol at −20 °C using a freeze cracking method, as described in [36]. Embryos were incubated for 45 min at room temperature with a mouse antitubulin antibody (1:100 clone DM1a; Sigma-Aldrich), rinsed, and then incubated with a secondary Dylight488-conjugated donkey anti-mouse antibody (1:500; Jackson ImmunoResearch Laboratories). DNA was stained with Hoechst 33342 (Sigma-Aldrich). Embryos were visualized using a confocal microscope (SP5 [Leica] or LSM710 [Zeiss]), and acquired images were processed with Image J software. Single confocal planes are shown in S1 Fig.

### Statistical analysis

Statistical analyses were performed using R (version 3.2.2). All R scripts are available upon request.

#### Mapping characteristics on the phylogenetic tree

A phylogenetic tree of 42 nematode species has previously been published [27]. To map the evolution of morphological characteristics on this phylogeny, we first obtained the mean value per species as an average of all strains. Ancestral states were estimated using the Brownian motion model and graphically mapped (contMap function in the R package Phytools 0.5–20).

#### Evolution of quantitative traits

Variations between species and between strains of the same species were modeled using a Bayesian method (fitBayes, R package Phytools [58,59]). The species average was then calculated, and the data were fit to the Ornstrein—Uhlenbeck model and the Brownian motion model using geiger R package [31]. *P*-values for model comparison were computed using likelihood ratio tests. Although the entire phylogeny of 42 species was analyzed for most traits, only the *Caenorhabditis* genus was used to analyze traits related to spindle oscillations, because they are specific to this monophyletic group.

#### Pairwise correlations accounting for phylogeny

Phylogenetic independent contrasts were performed on morphological values using the same nematode phylogeny, as implemented in the APE [60] or caper R packages (https://CRAN.R-project.org/package=caper).

#### Matrix of correlation

When correlating two traits measured in different species, nonrandom association can result from conservation since the common ancestor. Data points are not independent, and closely related species tend to have similar trait values. Methods have been proposed to correct for such phylogenetic inertia (e.g., phylogenetically independent contrasts [61] in ape R package or phylogenetic generalized least squares in the caper R package). Because intra-species variation is high in our dataset, we explored the range of correlations that can be obtained by randomly sampling one strain per species and then computing the correlation corrected for phylogenetic inertia on each subsample. We obtained 1,000 such correlations and statistically assessed these correlations. We obtained similar results using the average value of the parameter per species (S6 Fig). We also explored the possibility that imprecise measurements may prevent the detection of correlation between two variables (S7 Fig). We introduced up to 10% error in measurements and demonstrated that this did not prevented us from detecting an existing, even weak correlation. We are therefore confident that the absence of correlation is real in our dataset.

#### Principal component analysis

PCA highlights strong patterns in a multivariate dataset by identifying a space of principal components, which are independent combinations of parameters that better describe the variance of the data. PCA was performed using ade4 (version 1.7–4).

## Supporting information

S1 Fig Immunofluorescence of anaphase spindles in a subset of species. Immunofluorescent staining of fixed anaphase embryos showing microtubules (tubulin, green) and DNA (blue). Scale bar: 10 μm. Anterior is to the left. Light gray, species in the *Elegans* group; dark gray, species in the non-*Elegans* group; blue, species belonging to other genera.
(EPS)

S2 Fig Intra- and inter-species variations in cellular traits. (Left) Phylogeny of the species used in this study with number of strains per species shown in parentheses. *Caenorhabditis* non-*Elegans* species are shown in dark gray, *Elegans* group species are in light gray, other genera species are in blue. (Right) Scatter plot showing intra-species variations in cell length (A), asymmetry of division (B), spindle displacement (C), and spindle elongation fold (D). Each dot represents a strain, and the box and whiskers represent the median, quartiles, and range. Underlying numerical values are shown in S4 Table.
(EPS)

S3 Fig Variation in cell size, cell shape, and cell asymmetry. (A) Schematic representation of an embryo, showing the parameters associated with cell size, cell asymmetry, and cell shape. (B-D) Cell aspect ratio and cell asymmetry relative to the cell length for each strain (non-*Caenorhabditis* species in blue, *Caenorhabditis* strains in black, *C*. *elegans* N2 lab strain in red). Underlying numerical values are shown in S4 Table. (B) Cell aspect ratio corresponds to the ratio between cell length and cell width. (C) Relative cell size asymmetry corresponds to the ratio between the AB and P1 cell length. (D) The absolute position of the division plane represents the length of the P1 cell in microns.
(EPS)

S4 Fig Principal component analysis. PCA for all strains and parameters, excepted parameters related to spindle oscillations. Strains are colored according to their climate of origin (see also S1 Table). Underlying numerical values are shown in S8 Table. PCA, principal component analysis.
(EPS)

S5 Fig Orientation of parameter changes across species. (A-D) Phylogenetic trees colored according to the mean *value* per species for spindle elongation fold (A), cell length (B), amplitude of posterior oscillations (C), and final length of the spindle (D).
(EPS)

S6 Fig Pairwise correlation of traits using mean values per species. Covariation between pairs of parameters using the average value of the parameter per species. Color scale shows similarity scores and black outlined squares indicate significant correlations after correction for multiple testing (adjusted *p*-value < 0.05). *P*-values are shown in S7 Table. For parameters associated with spindle oscillations, only strains of the *Caenorhabditis* genus undergoing spindle oscillations were included.
(EPS)

S7 Fig Simulation of pairwise correlations when errors are added in the measurements. Graphs represent the frequency of correlations that are calculated between two variables for 1,000 simulations when 1%, 5%, or 10% of error are introduced in the data. The red bar corresponds to the initial correlation between the two variables. (A) For two variables showing an initial strong correlation (R = 0.8), we found that the correlation was still significant after introducing a 10% error rate. (B) For two variables showing an initial weak correlation (R = 0.2, *p*-value = 0.026), we found that the correlation between the two variables was lost when 10% of error was added to the data in only 83 cases out of 1,000, while 1% and 5% of error did not change the correlation between the variables. An error of 10% is a drastic change according to our own measurements. For instance, for an embryo 50 μm long, an error of 10% would correspond to a discrepancy of 38 pixels, which cannot really happen. From this, we concluded that errors in measurements do not prevent us from detecting an existing, even weak correlation, and we are therefore confident that the absence of correlation is real in our dataset.
(EPS)

S1 Table List of strains analyzed in this study. (XLSX)

S2 Table List of parameters measured from the DIC recordings. (DOCX)

S3 Table Data used to generate the manuscript Fig 1B and 1C. (XLSX)

S4 Table List of parameter values used in this study. (XLSX)

S5 Table Data used to generate the manuscript Fig 2A. (XLSX)

S6 Table Analysis of trait distribution. (XLSX)

S7 Table Data used to generate the manuscript Fig 3D and S6 Fig. (XLSX)

S8 Table Data used to generate the manuscript Fig 4A and 4B. (XLSX)

S9 Table Brownian motion vs. Ornstrein—Uhlenbeck model for each parameter. (XLSX)

## Abbreviations

CGC: Caenorhabditis Genetic Center
DIC: differential interference contrast
FDR: False Discovery Rate
NCC: nuclei centrosome complex
PCA: principal component analysis
