# Supplementary material for: Evolution of mitotic spindle behavior during the first asymmetric embryonic division of nematodes
Source: PLoS Biol. 2018 Jan 22;16(1):e2005099. doi: 10.1371/journal.pbio.2005099 (PMC5794175; doi:10.1371/journal.pbio.2005099)
Supplement: S2 Table — (DOCX) [file pbio.2005099.s009.docx]

| Measured parameters | Unit | Manual or automatic measurement |
| --- | --- | --- |
| Cell length | Micron | Manual |
| Cell width | Micron | Manual |
| Cell asymmetry | Ratio length anterior / posterior cell |  |
| Division plane | Micron | Length of the posterior P1 cell |
| Cell aspect ratio | Ratio Cell length/Cell width |  |
| Initial spindle length | Micron | Manual from curves of spindle length over time generated automatically |
| Final spindle length | Micron | Manual from curves of spindle length over time generated automatically |
| Spindle elongation speed | Micron/second | Manual from curves of spindle length over time generated automatically |
| Spindle elongation fold | Ratio Final spindle length/Initial spindle length |  |
| Initial spindle position | Micron | Automatic (Most anterior position of the center of the spindle relative to the center of the cell) |
| Final spindle position | Micron | Automatic (Position of the center of the spindle at the end of mitosis relative to the center of the cell) |
| Spindle displacement | Micron | Automatic (initial position to the final position) |
| Maximum amplitude of oscillations for the anterior centrosome | Micron | Automatic (larger peak to valley) |
| Maximum amplitude of oscillations for the posterior centrosome | Micron | Automatic (larger peak to valley) |
| Frequency of oscillations for the anterior centrosome | mHz | Automatic (mean of the frequencies) |
| Frequency of oscillations for the posterior centrosome | mHz | Automatic (mean of the frequencies) |
| Duration of oscillations for the anterior centrosome | Second | Semi-automatic (from curves obtained automatically, the onset and end of oscillations have been assigned manually) |
| Duration of oscillations for the posterior centrosome | Second | Semi-automatic (from curves obtained automatically, the onset and end of oscillations have been assigned manually) |
| Asymmetry of oscillations | Ratio (amplitude of the posterior centrosome/anterior centrosome) |  |
| Mean amplitude of oscillations for the anterior centrosome | Micron | Automatic (mean of each oscillation amplitude) |
| Mean amplitude of oscillations for the posterior centrosome | Micron | Automatic (mean of each oscillation amplitude) |
| Asymmetry of oscillations_mean | Ratio (mean amplitude of the posterior centrosome/anterior centrosome) |  |

**Table S2**: list of parameters measured from the DIC recordings
